# Characterization, isotherm and kinetic data for adsorption of Congo red and 2-naphthol on different bamboo hydrochars

**DOI:** 10.1016/j.dib.2018.04.066

**Published:** 2018-04-24

**Authors:** Yin Li, Arun Meas, Shengdao Shan, Ruiqin Yang, Xikun Gai, Hongpeng Wang, Nyamkhand Tsend

**Affiliations:** a Zhejiang Provincial Key Lab for Chemical and Biological Processing Technology of Farm Product, School of Biological and Chemical Engineering, Zhejiang University of Science and Technology, Hangzhou 310023, Zhejiang, China; b Key Laboratory of Recycling and Eco-treatment of Waste Biomass of Zhejiang Province, Zhejiang University of Science and Technology, Hangzhou 310023, Zhejiang, China; c Department of Agroindustry, Faculty of Agriculture and Food Processing, Mean Chey University, Sereisophoan 01252, Banteay Meanchey, Cambodia

## Abstract

Hydrochars were prepared using bamboo sawdust as raw material through hydrothermal carbonization with the present of acid or alkali in the medium and applied to remove Congo red and 2-naphthol from aqueous solutions. This data article provides information on FTIR and SEM profiles of the bamboo hydrochars, and the equation fitting results of the adsorption isotherms and kinetics for the two organics. The FTIR spectra show the differences of functional groups on the hydrochars with different process conditions. The SEM images show the surface morphology of selected hydrochars. Freundlich equation is slightly better than Langmuir model for the correlation of adsorption isotherms for both Congo red and 2-naphthol. Correlation coefficients from the pseudo-second order equation are greater than those of the pseudo-first order equation for both the organics on selected hydorchars.

**Specifications Table**

**Table:** 

Subject area	*Chemical Engineering*
More specific subject area	*Adsorption*
Type of data	*Table, image, figure*
How data was acquired	*The adsorption amount of Congo red and 2-naphthol by the hydrochars (Q*_*e*_*) was determined based on the subtraction of the initial and final concentration of the organics.*
	*FTIR: Bruker Vertex 70 FT-IR spectrometer*
	*SEM: Hitachi S3700 scanning electron microscope*
Data format	*Analyzed*
Experimental factors	*Bomboo hydrochars were grounded evenly for characterization.*
Experimental features	*The adsorption of Congo red and 2-naphthol on bamboo hydrochars.*
Data source location	*Zhejiang University of Science and Technology, Hangzhou, China*
Data accessibility	*Within this article*
Related research article	*The associated research article related to this data is**[1]*.

**Value of the data**

• The data shows detailed FTIR spectra of the bamboo hydrochars and provides detailed surface features of selected hydrochars.
• The isotherm fitting data gives information for modeling the capacity and explaining the mechanism for the adsorption of Congo red and 2-naphthol by the hydrochars.
• The kinetic fitting results will be useful for modeling and predicting the adsorption rate and rate-limiting step of Congo red and 2-naphthol on the hydrochars.

## 1 Data

**Fig. 1:**
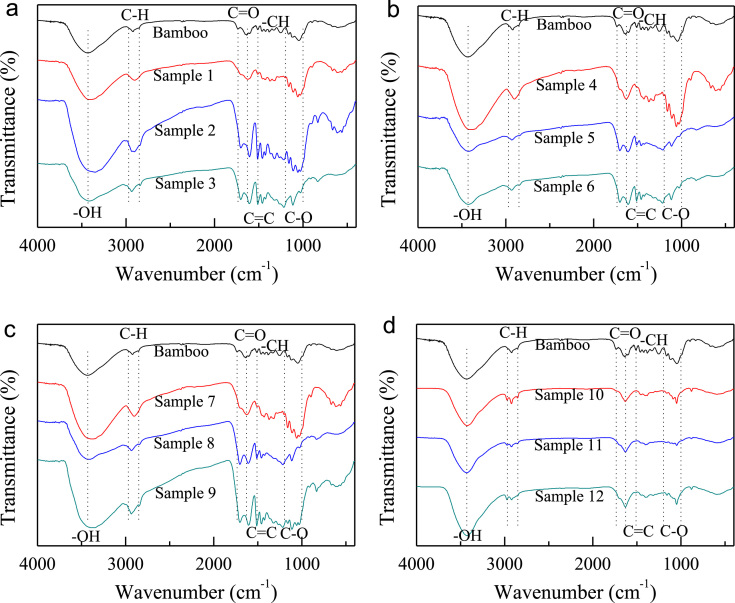
FTIR spectra of (a) bamboo sawdust and sample 1–3; (b) bamboo sawdust and sample 4–6; (c) bamboo sawdust and sample 7–9; and (d) bamboo sawdust and sample 10–12.

**Fig. 2:**
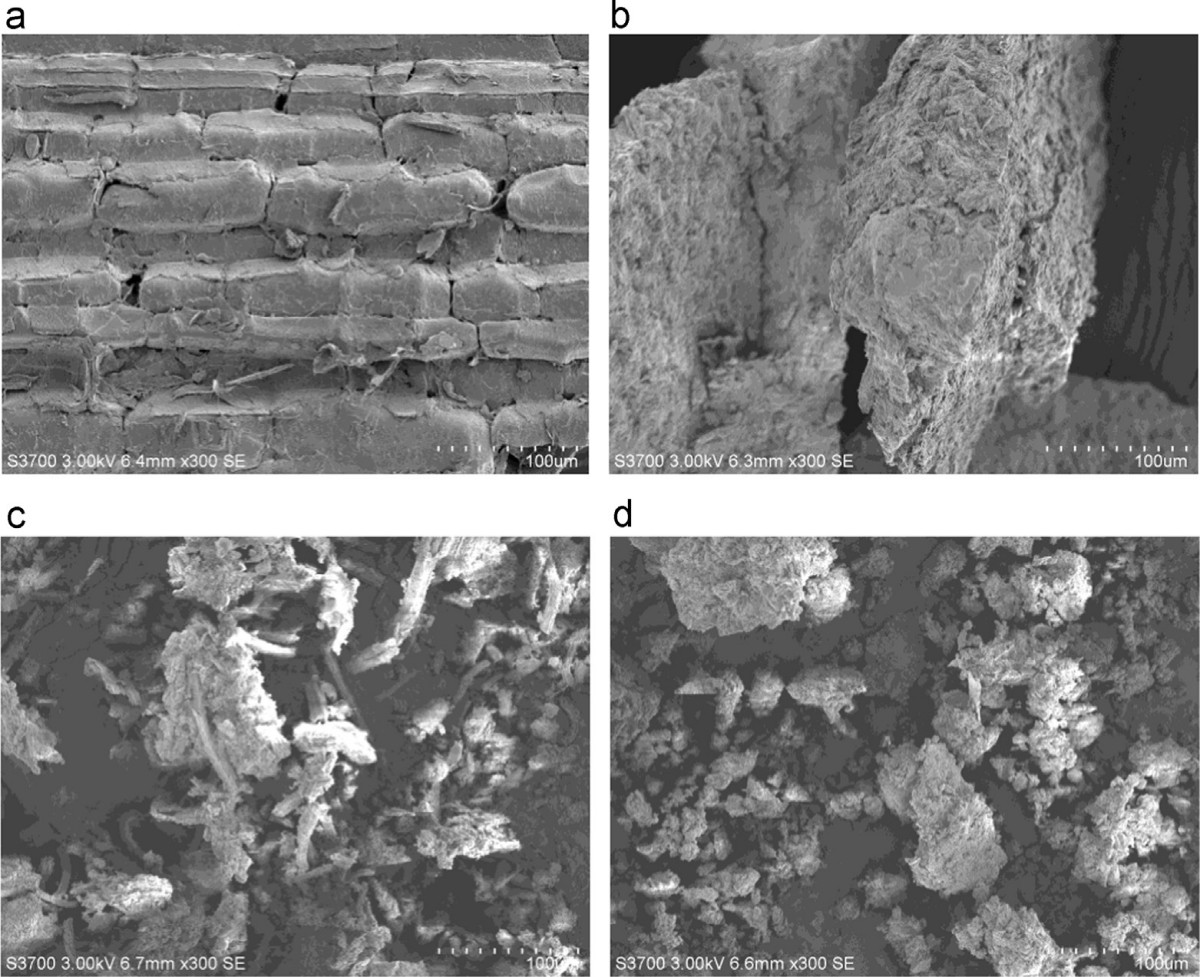
SEM images of (a) bamboo sawdust, (b) Sample 10, (c) Sample 11 and (d) Sample 12.

FTIR spectra of the bamboo hydrochars are shown in Fig. 1. Fig. 2. presents SEM images of selected hydrochars as well as the raw material bamboo sawdust. The parameters from Langmuir and Freundlich isotherm equations together with the correlation coefficients for Congo red and 2-naphthol at 298 K are summarizes in Table 1, and those on selected hydrochars at 298, 308 and 318 K are listed in Table 2. Kinetic parameters as well as the correlation coefficients for Congo red and 2-naphthol on selected hydrochars at 298 K are presented in Table 3.

**Table 1:** Langmuir and Freundlich isotherm parameters and correlation coefficients for the adsorption of Congo red and 2-naphthol at 298 K.

Adsorbate	Hydrochar sample	Langmuir isotherm			Freundlich isotherm		
		*Q*_m_ (mg/g)	*K*_L_ (mL/mg)	R^2^	*K*_F_ (mg/g)(mL/mg)^1/n^	1/n	R^2^
Congo red	Sample 1	43.02	6.67	0.988	47.13	0.430	0.994
	Sample 2	46.34	9.39	0.991	52.99	0.377	0.998
	Sample 3	37.80	13.45	0.975	43.60	0.313	0.991
	Sample 4	20.50	12.06	0.989	22.59	0.301	0.994
	Sample 5	14.04	16.17	0.995	15.21	0.239	0.990
	Sample 6	17.88	7.91	0.942	19.50	0.384	0.966
	Sample 7	37.65	6.81	0.983	41.16	0.423	0.992
	Sample 8	29.78	5.92	0.969	31.77	0.445	0.988
	Sample 9	22.23	7.63	0.953	24.15	0.389	0.976
	Sample 10	220.73	4.76	0.990	228.18	0.489	0.998
	Sample 11	234.91	4.69	0.980	243.79	0.496	0.995
	Sample 12	255.61	5.47	0.994	273.50	0.470	0.999
2-naphthol	Sample 1	51.32	3.73	0.997	51.41	0.551	0.999
	Sample 2	48.38	1.92	0.983	38.48	0.661	0.991
	Sample 3	44.68	3.12	0.994	42.50	0.582	0.997
	Sample 4	54.82	4.84	0.991	58.04	0.500	0.996
	Sample 5	50.57	9.66	0.959	58.93	0.384	0.974
	Sample 6	62.02	5.29	0.991	67.65	0.492	0.997
	Sample 7	67.17	3.30	0.998	66.20	0.584	0.999
	Sample 8	95.26	2.93	0.986	92.39	0.618	0.996
	Sample 9	68.44	4.48	0.988	72.78	0.527	0.999
	Sample 10	322.56	1.26	0.992	213.81	0.745	0.993
	Sample 11	272.20	3.66	0.985	268.69	0.546	0.997
	Sample 12	302.78	1.89	0.977	240.95	0.670	0.981

**Table 2:** Langmuir and Freundlich isotherm parameters and correlation coefficients for the adsorption of Congo red on Sample 12, and 2-naphthol on Sample 11 at 298 K, 308 K and 318 K.

Adsorbate	Hydrochar sample	T (K)	Langmuir model			Freundlich model		
			*Q*_m_ (mg/g)	*K*_L_ (mL/mg)	R^2^	K_F_ (mg/g) (mL/mg)^1/n^	1/n	R^2^
Congo red	Sample 12	298	255.61	5.47	0.994	273.50	0.470	0.999
		308	375.50	2.89	0.983	351.06	0.597	0.995
		318	635.16	1.32	0.999	445.49	0.766	0.997
2-naphthol	Sample 11	298	272.20	3.66	0.985	268.69	0.546	0.997
		308	327.07	2.15	0.984	274.52	0.648	0.991
		318	299.60	1.96	0.985	241.44	0.663	0.993

**Table 3:** Kinetic parameters for adsorption of Congo red on sample 12 and 2-naphthol on sample 11 at 298 K.

Adsorbate	Hydrochar sample	Pseudo-first-order equation			Pseudo-second-order equation		
		*q*_e_ (mg/g)	*k*_1_ (1/min)	R^2^	*q*_e_ (mg/g)	*k*_2_ (mg/(g.min))	R^2^
Congo red	Sample 12	169.59	0.564	0.959	174.42	0.0030	0.969
2-naphthol	Sample 11	156.50	0.614	0.938	163.62	0.0026	0.978

## 2 Experimental design, materials, and methods

### 2.1 Preparation of the bamboo hydrochars

Bamboo hydrochars were prepared through acid-assisted or two-stage hydrothermal treatment. The feed solutions in acid-assisted hydrothermal treatment for the preparation of hydrochar sample 1–9 are 5 wt% HNO_3_, 10 wt% HNO_3_, 15 wt% HNO_3_, 5 wt % H_2_SO_4_, 10 wt % H_2_SO_4_, 15 wt % H_2_SO_4_, 5 wt % H_3_PO_4_, 10 wt % H_3_PO_4_ and 15 wt % H_3_PO_4_, respectively. The feed solutions in two-stage hydrothermal carbonization process for the production of hydrochar sample 10–12 are 5 wt % NaOH in the first stage, and 5 wt% HNO_3_, 5 wt % H_2_SO_4_ and 5 wt % H_3_PO_4_, respectively in the second stage.

In an acid-assisted HTC process, about 6.00 g of wet bamboo sawdust was mixed with 36.0 mL of acid solution in a 100 mL autoclave reactor with an internal Teflon insert, and the concentrations of the acid solution were from 5 to 15 wt%. The reactor was sealed and heated at 200 °C for 3 h, then cooled down to room temperature in cold water. The solid product was collected by vacuum filtration, washed with deionized water until pH of the washed water was around 7.0, and dried at 100 °C for 12 h.

In a two-stage hydrothermal treatment process, 6.00 g of bamboo sawdust was added into 36.0 mL of 5 wt% NaOH solution in a Teflon lined autoclave reactor and the mixture was held at 200 °C for 3 h. The reactor was then cooled down and the solid product was collected, washed and dried. 6 mL of 5 wt% acid solution was mixed with per 1 g of the obtained solid material, the mixture was loaded in a Teflon lined autoclave reactor and hydrochars were prepared through an acid-assisted HTC process described above.

### 2.2 Characterization of the hydrochars

Fourier transform infrared spectroscopy (FTIR) spectra of all hydrochar samples were recorded by a Bruker Vertex 70 FT-IR spectrometer. Hitachi S3700 scanning electron microscope (SEM) was used to study the surface morphology of the hydrochars.

### 2.3 Adsorption isotherms

Hydrochars (0.0100 g) were placed in 30 mL of Congo red or 2-naphthol solutions with known initial concentrations (*C*_0_) in capped glass flasks. The flasks were shaken at 170 rpm and set temperatures in a TS-1102TENSUC shaker for 8 h. The suspensions were then filtered, and the equilibrium concentrations *C*_e_ (mg/mL) of Congo red and 2-naphthol in the supernatant were determined using a UV–vis spectrophotometer at wavelength of 499 nm and 274 nm respectively. Adsorption capacities *Q*_e_ (mg/g) were calculated from the difference between equilibrium concentrations and initial ones:

(1) $$Q_{e}=(C_{0}-C_{e})\cdot V/m$$

Where *V* (mL) is the volume of the suspension and *m* (g) is the adsorbent dry mass.

The experimental adsorption isotherm data was analyzed using the Langmuir and Freundlich models:

Langmuir model:

(2) $$Q_{e}=\frac{Q_{m}K_{L}C_{e}}{1+K_{L}C_{e}}$$

Freundlich model:

(3) $$Q_{e}=K_{F}{C_{e}}^{1/n}$$

Where *Q*_*m*_ (mg/g) is the quantity adsorbed per gram of adsorbent to give a complete monolayer, *K*_*L*_ (mL/mg) is the Langmuir constant related to capacity and rate of adsorption [2]. *K*_*F*_ ((mg/g)(mL/mg)^1/n^) and 1/n are the Freundlich constants indicating relative adsorption capacity and favorability of the adsorption, respectively [3].

### 2.4 Adsorption kinetics

0.0100 g of the selected hydrochars was added into 30 mL of 0.5 mg/mL Congo red or 2-naphthol solution in a capped glass flask The flasks were shaken at 170 rpm and 298 K, the contact time *t*(min) was ranging from 5 to 300 min, and the concentrations of Congo red and 2-naphthol in the suspensions at *t C*_t_ (mg/mL) were determined after filtration. Adsorption capacities at *t*, *Q*_t_ (mg/g) were calculated by:

(4) $$Q_{t}=(C_{0}-C_{t})\cdot V/m$$

The adsorption kinetic data was analyzed by the pseudo-first order and pseudo-second order models:

Pseudo-first order equation:

(5) $$Q_{t}=q_{e}(1-e^{-k_{1}t})$$

Pseudo-second order equation:

(6) $$Q_{t}=\frac{tq_{e}^{2}k_{2}}{tk_{2}q_{e}+1}$$

Where *k*_1_ (1/min) and *k*_2_ (g/(mg min)) are the pseudo-first-order rate and the pseudo-second-order rate constants, *q*_e_ (mg/g) is the calculated adsorbed quantity at equilibrium.
